# Conflict-affected displaced persons need to benefit more from HIV and malaria national strategic plans and Global Fund grants

**DOI:** 10.1186/1752-1505-4-2

**Published:** 2010-01-29

**Authors:** Paul B Spiegel, Heiko Hering, Eugene Paik, Marian Schilperoord

**Affiliations:** 1 Public Health and HIV Section, United Nations High Commissioner for Refugees, Geneva, Switzerland

## Abstract

**Background:**

Access to HIV and malaria control programmes for refugees and internally displaced persons (IDPs) is not only a human rights issue but a public health priority for affected populations and host populations. The primary source of funding for malaria and HIV programmes for many countries is the Global Fund to Fight AIDS, Tuberculosis and Malaria (Global Fund). This article analyses the current HIV and malaria National Strategic Plans (NSPs) and Global Fund approved proposals from rounds 1-8 for countries in Africa hosting populations with refugees and/or IDPs to document their inclusion.

**Methods:**

The review was limited to countries in Africa as they constitute the highest caseload of refugees and IDPs affected by HIV and malaria. Only countries with a refugee and/or IDP population of ≥ 10,000 persons were included. NSPs were retrieved from primary and secondary sources while approved Global Fund proposals were obtained from the organisation's website. Refugee figures were obtained from the United Nations High Commissioner for Refugees' database and IDP figures from the Internal Displacement Monitoring Centre. The inclusion of refugees and IDPs was classified into three categories: 1) no reference; 2) referenced; and 3) referenced with specific activities.

**Findings:**

A majority of countries did not mention IDPs (57%) compared with 48% for refugees in their HIV NSPs. For malaria, refugees were not included in 47% of NSPs compared with 44% for IDPs. A minority (21-29%) of HIV and malaria NSPs referenced and included activities for refugees and IDPs. There were more approved Global Fund proposals for HIV than malaria for countries with both refugees and IDPs, respectively. The majority of countries with ≥10,000 refugees and IDPs did not include these groups in their approved proposals (61%-83%) with malaria having a higher rate of exclusion than HIV.

**Interpretation:**

Countries that have signed the 1951 refugee convention have an obligation to care for refugees and this includes provision of health care. IDPs are citizens of their own country but like refugees may also not be a priority for Governments' NSPs and funding proposals. Besides legal obligations, Governments have a public health imperative to include these groups in NSPs and funding proposals. Governments may wish to add a component for refugees that is additional to the needs for their own citizens. The inclusion of forcibly displaced persons in funding proposals may have positive direct effects for host populations as international and United Nations agencies often have strong logistical capabilities that could benefit both populations. For NSPs, strong and concerted advocacy at global, regional and country levels needs to occur to successfully ensure that affected populations are included in their plans. It is essential for their inclusion to occur if we are to reach the stated goal of universal access and the Millennium Development Goals.

## Background

Forcibly displaced persons, such as refugees and internally displaced persons (IDPs) have fled their dwellings due to violent conflict and seek protection and refuge away from their home. They often live on marginal land in rural areas or in overcrowded urban environments with limited or no access to public services. The infrastructure among their host communities is often weak and overwhelmed by the additional demands of these displaced persons. Human immunodeficiency virus (HIV) and malaria are often major public health issues among these groups. For example, almost two thirds of refugees, IDPs and other persons of concern to the United Nations High Commissioner for Refugees (UNHCR) live in areas where malaria is a leading cause of morbidity and mortality. Furthermore, many displaced persons are situated in Africa, where morality and morbidity is due to HIV and AIDS is often very high.

Access to HIV and malaria control programmes for forcibly displaced persons is not only a protection and human rights issue but a public health priority for both affected populations and their surrounding host populations[1]. Whenever possible, parallel services for refugees and IDPs should be avoided; it is more cost effective and equitable to integrate these groups into existing services available to their host populations. To do this, Governments must include refugees and IDPs into their national strategic plans (NSPs) as well as funding proposals.

The primary source of funding for malaria and HIV programmes for many countries hosting refugees and IDPs is the Global Fund to Fight AIDS, Tuberculosis and Malaria (Global Fund). Global Funds grants have increased from US$1.7 billion in January 2002[2] to US$ 2.75 billion for a two-year target in Round 8[3].

The objective of this article is to analyse the current HIV and malaria NSPs as well as approved Global Fund proposals with HIV and/or malaria components from rounds 1-8 for countries in Africa hosting populations of ≥ 10,000 refugees and/or IDPs and to document their inclusion.

## Methods

The review was limited to countries in Africa as they constitute the highest caseload of refugees and IDPs affected by malaria and HIV. Only countries with a refugee and/or IDP population of ≥ 10,000 persons were included. This inclusion criterion was applied to each country for a period of 10 years from 1998 to 2008 for the review of NSPs and for the year of the Global Fund proposal submission for rounds 1 to 8 from 2002 to 2008 to adjust for population changes over time. Only accepted Global Fund proposals with a malaria and/or HIV component were included. Algeria, Libya and Egypt are included for the review of HIV NSPs and Global Fund proposals but excluded from the malaria component as malaria is not prevalent in those countries.

NSPs for malaria and HIV were retrieved from primary sources (e.g. Government websites and contact persons) as well as secondary sources (e.g. Roll Back Malaria and UNAIDS Secretariat). Additionally, UNHCR staff located in-country contacted UN Theme Groups and Governments to locate plans. Approved proposals from the Global Fund were obtained from the organisation's website.

UNHCR's database was used to obtain population figures for refugees[4]. IDP population sizes were used from the Internal Displacement Monitoring Centre of the Norwegian Refugee Council[5]. Tuberculosis was excluded as refugees and IDPs are generally included in national tuberculosis programmes.

The inclusion of refugees and IDPs was classified into three categories: 1) No reference to any of the keywords was classified as "no mention"; 2) The mention of one or more of the keywords (see below) without specific reference to any activity, programme and/or funding directed at refugees and/or IDPs was classified as "reference"; 3) The mention of one or more keywords within the context of specific activities, programmes or funds being directed at refugees and/or IDPs was classified as "reference and activities".

The following keywords were selected for the review: refugee, internally displaced person, IDP, returnee, displaced person, and mobile person (excluding nomadic, semi-nomadic and migrant worker). The search term 'person' was replaced with 'people' and 'population' when appropriate. Singular and plural forms were searched. Returnees were classified as refugees. For French documents, the equivalent French keywords were used. The search of documents was carried out in two stages. Initially, every document was electronically searched for each of the keywords. This was followed by a thorough read-through of every document including those that did not reveal electronic search results.

## Findings

The number of countries with ≥ 10,000 refugees and IDPs varied according to the dates of the NSPs and approved Global Fund proposals. For the NSPs, there were 33 African countries with ≥ 10,000 refugees and 22 countries with ≥ 10,000 IDPs for HIV, and 30 countries with ≥ 10,000 refugees and 21 countries with ≥ 10,000 IDPs for malaria during the study period. For the approved Global Fund proposals, there were 33 African countries with ≥ 10,000 refugees and 19 countries with ≥ 10,000 IDPs for HIV, and 30 countries with ≥ 10,000 refugees and 18 countries with ≥ 10,000 IDPs for malaria during the study period. (See table 1)

**Table 1:** Inclusion of ≥10,000 refugees and/or IDPs in African countries in HIV and malaria National Strategic Plans and Global Fund approved proposals

National Strategic Plans	Assessed	%	No Mention	%	Reference	%	Reference with Activities	%
*HIV*								

Refugee (N = 33)	21	63.6%	10	47.6%	5	23.8%	6	28.6%

IDP (N = 22)	14	63.6%	8	57.1%	3	21.4%	3	21.4%

*Malaria*								

Refugee (N = 30)	15	50.0%	7	46.7%	5	33.3%	3	20.0%

IDP (N = 21)	9	42.9%	4	44.4%	3	33.3%	2	22.2%

Global Fund Approved Proposals, Rounds 1-8*	**Assessed**	**%**	**No Mention**	**%**	**Reference**	**%**	**Reference with Activities**	**%**

*HIV*								

Refugee (N = 33)	70	100.0%	43	61.4%	19	27.1%	8	11.4%

IDP (N = 19)	26	100.0%	16	61.5%	5	19.2%	5	19.2%

*Malaria*								

Refugee (N = 30)	53	100.0%	44	83.0%	3	5.7%	6	11.3%

IDP (N = 18)	24	100.0%	17	70.8%	4	16.7%	3	12.5%

* Multiple approved proposals from rounds 1-8 from the countries were included when relevant. All approved proposals were assessed.

More NSPs for HIV were found and assessed for both refugees and IDPs than for malaria. A majority of countries did not mention IDPs (57%) compared with 48% for refugees in their HIV NSPs. For malaria, refugees were not included in 47% of NSPs compared with 44% to IDPs. A minority (between 20-29%) of malaria and HIV NSPs that were assessed actually referenced and included activities for refugees and IDPs (see table 1). For those countries that mentioned malaria activities, the main interventions were distribution of long lasting insecticide treated bed nets, indoor residual spraying and outreach activities.

**Figure 1:**
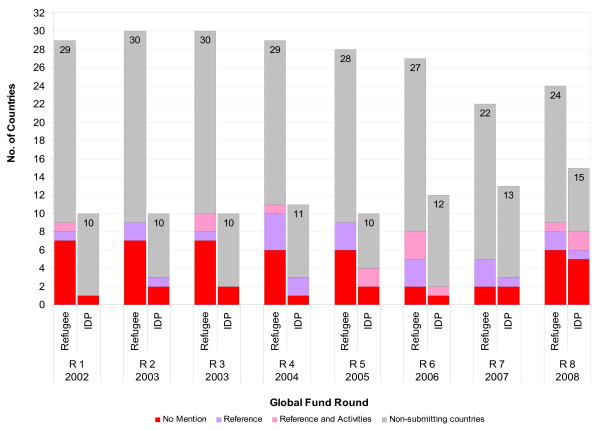
**Inclusion of refugees and/or IDPs in accepted Global Fund proposals with HIV component in African countries with ≥ 10,000 refugees and/or ≥ 10,000 IDPs**. Rounds 1-8 (2002-2008).

**Figure 2:**
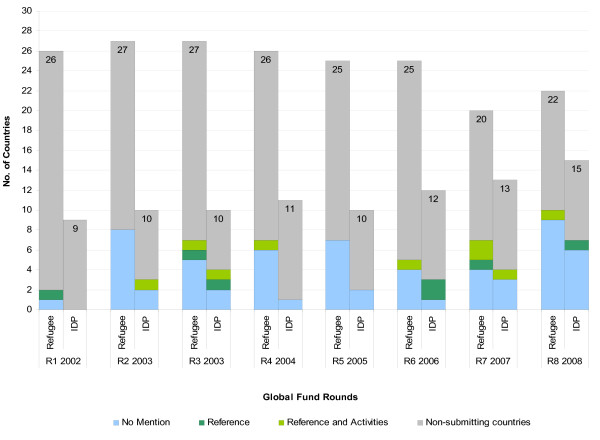
**Inclusion of refugees and/or IDPs in accepted Global Fund proposals with malaria component in African countries with ≥ 10,000 refugees and/or ≥ 10,000 IDPs - Rounds 1-8 (2002-2008)**.

There were more approved Global Fund proposals for HIV than malaria for countries with both refugees and IDPs, respectively. The majority of countries with ≥ 10,000 refugees and IDPs did not include these groups in their approved proposals (range: 61%-83%) with malaria having higher rate of exclusion than HIV. A minority of approved proposals referenced and had specific activities for refugees and IDPs with IDPs for HIV proposals having the highest inclusion at 19% (See figures 1 and 2).

Egypt and Sierra Leone were the only two countries that referenced and included similar activities for refugees in their HIV NSPs and Global Fund approved HIV proposals. Sudan is the only country that referred to and included specific malaria activities for both refugees and IDPs in its NSP and Global Fund approved proposals. Bednet distribution was the main activity listed in the plan and proposal for both groups. Uganda referred to IDPs and Tanzania to refugees in their malaria NSPs and approved Global Fund proposals but no specific activities were mentioned.

## Interpretation

The majority of African countries with ≥ 10,000 refugees and/or IDPs did not include them in their approved Global Fund proposals for malaria and for HIV. Furthermore, a large proportion of countries with ≥ 10,000 refugees and/or IDPs did not mention them in their malaria and HIV NSPs. This lack of inclusion occurred despite the fact that refugees and IDPs in most of these countries have been settled there for many years, and in some cases decades. Only a minority of those countries both referenced refugees and/or IDPs and specifically included activities in their NSPs and approved Global Fund proposals for malaria and HIV.

A Government's first inclination is to take care of its own citizens. Therefore, refugees will rarely if ever be a Government's first priority. However, those countries that have signed the 1951 refugee convention[6] have an obligation to care for refugees and this includes the provision of health care. IDPs are citizens of their own country. However, they are often oppressed by the Government in power and thus, like refugees, may also not be a priority for NSPs and funding proposals.

Besides legal obligations, Governments have a public health imperative to include refugees, IDPs and other groups, such as economic migrants, in their disease specific strategic plans and funding proposals. Communicable diseases do not respect borders and it is not effective public health policy to provide prevention and treatment programmes to only part of a population residing in the same geographical area.

Refugees and IDPs are often located in isolated and relatively inaccessible areas where Government infrastructure, systems and personnel are marginal. Government health interventions are often poorly implemented for nationals in these remote areas. The inclusion of forcibly displaced persons in funding proposals may have positive direct effects for the host populations as international and United Nations (UN) agencies operating in these locations often have strong logistical capabilities that could benefit all populations. Consequently, the equity of providing interventions to more remote areas of a country, a major problem in many nations where urban and peri-urban populations primarily benefit from such programmes, could be improved.

In many settings, refugee and IDPs compose only a small proportion of the total population of a country. Although they often live in inaccessible and remote areas, there are always surrounding populations from the country that live there as well. Therefore, the relative additional cost in including them in proposals and programmes is marginal, as Governments must also provide such interventions to their citizens already living in these areas. Governments may wish to consider the needs of their own populations first (including IDPs), and then add a component for refugees that is additional to the needs of their own citizens. In this way, concerns about using limited funds for persons other than one's own citizens are negated.

For NSPs, strong and concerted advocacy at global, regional and country levels needs to occur to successfully ensure that refugees and IDPs are included in national disease-specific plans. Improved coordination among Governments, the UN system and civil society during the planning and revision of national plans is sorely needed. The importance of their inclusion has grown considerably with the recent Global Fund Board's decision to move towards funding countries' NSPs in future rounds. Furthermore, since universal access for malaria and HIV control is a declared goal,[7,8] inclusion of displaced populations is a necessity if the world is to meet these aspirations. The same holds true for the Millennium Development Goals[9]. For malaria, regional meetings are planned to update the current national plans for 2011-2015. Effective advocacy during these meetings would be very useful. Unfortunately, we are not aware of a similar process for HIV NSPs.

Global Fund proposals are made by Country Coordinating Mechanisms (CCMs) that are composed of a wide variety of groups including Government, civil society, and the private sector. UN organisations are often part of the CCM as well. Although in many countries the CCM is dominated by the Government, all groups that constitute the CCM have an obligation to include all persons that reside in a country, and not just the country's citizens. Furthermore, the Global Fund's Technical Review Panel should be obliged to consider these groups in country proposals. The exclusion of the above mentioned groups will limit the effectiveness of the interventions no matter how technically sound the proposals are written for the rest of the population; in essence, proposals that do not consider these groups are not technically sound.

Recently, a small informal working group composed of the Global Fund and UN agencies was formed with the objective to examine how Global Fund monies could possibly be used to address different humanitarian contexts; the Global Fund was not created with this in mind. However, clearly there is a need. Humanitarian emergencies are not simply acute events of a short duration; most last for years and even decades. The divide between humanitarian and development funding is well known and has never been sufficiently addressed. Ultimately, however, the Global Fund is a country-driven process led by the CCMs. Thus, guidance and advocacy need to be directed at the country level. Positive examples include Sudan which has included specific activities for refugees, IDPs and returnees in their malaria NSPs as well as Global Fund proposals.

There are some limitations to our study. Not all NSPs for African countries with ≥ 10,000 refugees and/or IDPs were identified, despite in-country attempts to locate them. For those countries where plans were not found, it is unclear which countries do not have such plans or which were simply not accessible. Tuberculosis was not included in the study because of our experience that refugees, even in remote areas, have free access to Government tuberculosis programmes. We did not have access to those countries that submitted proposals to the Global Fund that may have included conflict-affected persons but were rejected.

Governments, development agencies and donors must recognise the human right and public health imperative as well as the long-term implications of not including persons displaced by conflict into NSPs and funding proposals. In 2001, the UN General Assembly adopted the Declaration of Commitment on HIV/AIDS "recognizing that populations destabilized by armed conflict, humanitarian emergencies and natural disasters, including refugees, internally displaced persons, and in particular women and children, are at increased risk of exposure to HIV infection" and that there is a need to "implement national strategies that incorporate HIV/AIDS awareness, prevention, care and treatment elements into programmes or actions that respond to emergency situations..."[10]. The Political Declaration on HIV/AIDS in 2006 reaffirmed these commitments in the context of achieving universal access to HIV prevention, treatment, care and support for vulnerable groups, including refugees and internally displaced persons[8]. The 2008 Global Malaria Action Plan unambiguously refers to populations affected by emergencies and displacement, and calls for their inclusion into malaria control programmes[7].

This study shows that at present these calls for action are not being heeded. Besides including conflict-affected populations that have been displaced for long periods of time into NSPs and funding proposals, Governments and other actors should ensure that contingency plans for such occurrences are included in these plans and proposals. This inclusion will allow for the flexibility to prioritise and transfer funds to these affected populations in a short period of time if needed. Donors should ensure that such a mechanism exists in their regulations to allow for such contingencies. A concerted effort by numerous actors including Governments, UN agencies, international organisations, donors, civil society and the private sector, that bridge both the humanitarian and development worlds, is necessary if we are to include conflict affected populations in NSPs and funding proposals and reach the lofty aspirations of universal access and the Millennium Development Goals.

## Competing interests

The authors declare that they have no competing interests.

## Authors' contributions

PS developed concept of paper, participated in the analysis, participated in drafting of the manuscript.

HH participated in the research, analysis and drafting of the paper.

EP participated in the research, analysis and drafting of the paper.

MS participated in the analysis and drafting of the paper.

All authors read and approved the final manuscript.
